# Dynamic LED light versus static LED light for depressed inpatients: results from a randomized feasibility trial

**DOI:** 10.1186/s40814-019-0548-9

**Published:** 2020-01-15

**Authors:** Carlo Volf, Anne Sofie Aggestrup, Signe Dunker Svendsen, Torben Skov Hansen, Paul Michael Petersen, Carsten Dam-Hansen, Ulla Knorr, Ema Erkocevic Petersen, Janus Engstrøm, Ida Hageman, Janus Christian Jakobsen, Klaus Martiny

**Affiliations:** 1 NID GROUP, Copenhagen Affective Disorder Research Center (CADIC), Psychiatric Center Copenhagen, University of Copenhagen, Rigshospitalet, Copenhagen, Denmark; 2 Chromaviso A/S, Aarhus, Denmark; 3 Department of Photonics Engineering, Technical University of Denmark, Kongens Lyngby, Denmark; 4 Copenhagen Trial Unit, Centre for clinical intervention research, Rigshospitalet, Copenhagen, Denmark; 5 Mental Health Services, Capital Region, Copenhagen, Denmark; 6 Department of Regional Health Research, The Faculty of Health Sciences, University of Southern Denmark, Odense, Denmark; 7 Department of Cardiology, Holbæk Hospital, Holbæk, Denmark

**Keywords:** Major depressive disorder, Bipolar disorder, Sleep, Lighting, Circadian, Light, Hospitals, Chronotherapy, Randomisation, RCT, Depression, Architecture

## Abstract

**Background:**

Retrospective studies conducted in psychiatric wards have indicated a shorter duration of stay for depressed inpatients in bright compared to dim daylight-exposed rooms, pointing to a possible antidepressant effect of daylight conditions. Dynamic LED lighting, aiming to mimic daylight conditions, are currently been installed in several hospitals, but their feasibility is poorly investigated.

**Methods:**

To investigate the feasibility of these systems, we developed and installed a LED-lighting system in four rooms in a psychiatric inpatient ward. The system could function statically or dynamically regarding light intensity and colour temperature. The system consisted of (A) a large LED luminaire built into the window jamb mimicking sunlight reflections, (B) two LED light luminaires in the ceiling and (C) a LED reading luminaire. In the static mode, the systems provided constant light from A and B. In the dynamic mode, the system changed light intensity and colour temperature using A, B and C. Patients with unipolar or bipolar depression were randomised to dynamic or static LED lighting for 4 weeks, in addition to standard treatment. Primary outcome was the rate of patients discontinuing the trial due to discomfort from the lighting condition. Secondary outcomes were recruitment and dropout rates, visual comfort, depressive symptoms and suicidal ideation.

**Results:**

No participants discontinued due to discomfort from the LED lighting. Recruitment rate was 39.8%, dropout from treatment rates were 56.3% in the dynamic group and 33.3% in the static group. 78.1% in the dynamic group were satisfied with the lighting compared with 71.8% in the static group. Discomfort from the light (glare) was reported by 11.5% in the dynamic group compared to 5.1% in the static group. Endpoint suicidal scores were 16.8 (10.4) in the dynamic and 16.3 (14.9) in the static group. The lighting system was 100% functional. The light sensor system proved unstable.

**Conclusion:**

Dropout from treatment was high primarily due to early discharge and with a lack of endpoint assessments. The feasibility study has influenced an upcoming large-scale dynamic lighting efficacy trial where we will use a shorter study period of 3 weeks and with more emphasis on endpoint assessments. The lighting was well tolerated in both groups, but some found intensity too low in the evening. Thus, we will use higher intensity blue-enriched light in the morning and higher intensity amber (blue-depleted) light in the evening in the upcoming study. The light sensor system needs to be improved

**Trial registration:**

ClinicalTrials.gov: NCT03363529

## Background

The treatment of patients with severe depression, whether as part of a unipolar or bipolar disorder, still lacks efficacy. Patients are often discharged with residual depressive symptoms [1] making them at risk of relapse [2]. There has been no recent breakthrough in psychopharmacology or psychotherapy, and new methods are therefore needed. Chronotherapeutic treatments, including bright light therapy, have shown promising preliminary results in the treatment of both unipolar and bipolar depression [3–7]. Some studies have included both unipolar and bipolar depressed patients, but the results have not been reported separately, so we do not know whether light therapy works best in bipolar or unipolar depression. Light therapy has traditionally been based on lightboxes used in the morning for 30–60 min [8]. Light therapy seems to have relatively few adverse effects [9], and with a good ocular safety [10], but it probably has to be continued to maintain the antidepressant effect [11]. The recent discovery of the intrinsically photosensitive retinal ganglion cells (ipRGC) [12], with peak sensitivity around 480 nm, and the development of the light-emitting diode (LED) technology have made it clinically relevant and possible to tailor specific light intensities and spectral distributions throughout the 24-h day. This technical development has spurred an interest, in psychiatric settings, to move from lightbox-administered light therapy towards using integrated LED-lighting systems exposing patients to dynamic light therapy throughout the day. A few retrospective cohort studies based on daylight or LED light at inpatient wards have been carried out showing a shortened length of inpatient stay with brighter conditions [13–16]. Our group has recently investigated the impact of daylight in psychiatric inpatients and found a shorter stay in rooms located on the brighter south-east side of the building compared to rooms on the dimmer north-west side [17]. A recent randomised trial in post-stroke patients showed a strengthening of melatonin rhythmicity and improved mood with brighter light conditions [18, 19].

Currently, dynamic LED lighting is increasingly being installed in hospital settings attempting to mimic daylight conditions with temporally regulated intensity and spectral distribution. The aim is to strengthen circadian entrainment to stabilise the sleep-wake cycle, provide alertness and increase the antidepressant effect. However, the usability, acceptability, adverse effects and efficacy of these systems are poorly investigated.

This study aims to investigate the feasibility of a dynamic versus a static lighting condition delivered to patients with depressive disorders as a pre-study to a planned larger efficacy study with dynamic lighting. In this study, we chose to include both patients with unipolar and bipolar depression because we wanted to know whether patients with bipolar depression would switch to a hypomanic or manic state. We also generally need more data on light treatment in bipolar patients as this condition has been studied less than unipolar depression.

## Methods

We developed a new general lighting system using dynamic LED lighting installed in four single-bed patient rooms in a psychiatric inpatient ward. The system consisted of (A) a large LED panel built into the window jamb to mimic sunlight reflections, (B) two dynamic LED light fixtures in the ceiling and (C) one dynamic LED reading luminaire by the bed. Patients with a current depressive episode as part of a unipolar or a bipolar depression were randomised, in a 3:2 manner to either dynamic LED-lighting intervention (dynamic) or static LED-lighting intervention (static), for 4 weeks in addition to standard treatment. The randomisation was done automatically within the OpenClinica electronic Case Report Form (eCRF) system after entering of all eligibility criteria, and randomisation was thus concealed from investigators. The OpenClinica eCRf system was operated by the Copenhagen Trial Unit (CTU). We opted for 15 participants as we anticipated that this number would give indications of major problems with the lighting system, trial design, and adverse events. The randomisation with more participants into the dynamic group was chosen to increase the likelihood of finding serious adverse events in this group where we expected that the brighter light and a luminaire that could not be turned off might cause problems for the participants.

### Participant selection

The screening was done at the inpatient ward. Diagnoses were assessed by the Mini International Neuropsychiatric Interview (M.I.N.I.) [20]. Inpatients were consecutively asked to partake in the study. Inclusion criteria were age > 18 years, a major depressive episode as part of a unipolar or bipolar disorder according to the Diagnostic and Statistical Manual version IV (DSM-IV) [21], informed consent and speaks and understands the Danish language. Exclusion criteria were severe suicidality corresponding to a score > 2 on Hamilton depression rating scale item 3 or if the investigators were unsure of the degree of suicidality, current psychotic symptoms at time of inclusion, Young Mania Rating Scale (YMRS) > 7, fulfilling DSM-IV criteria for a current hypomanic or manic episode, or mandatory psychiatric treatment of any kind.

### Experimental protocol

**Fig. 1:**
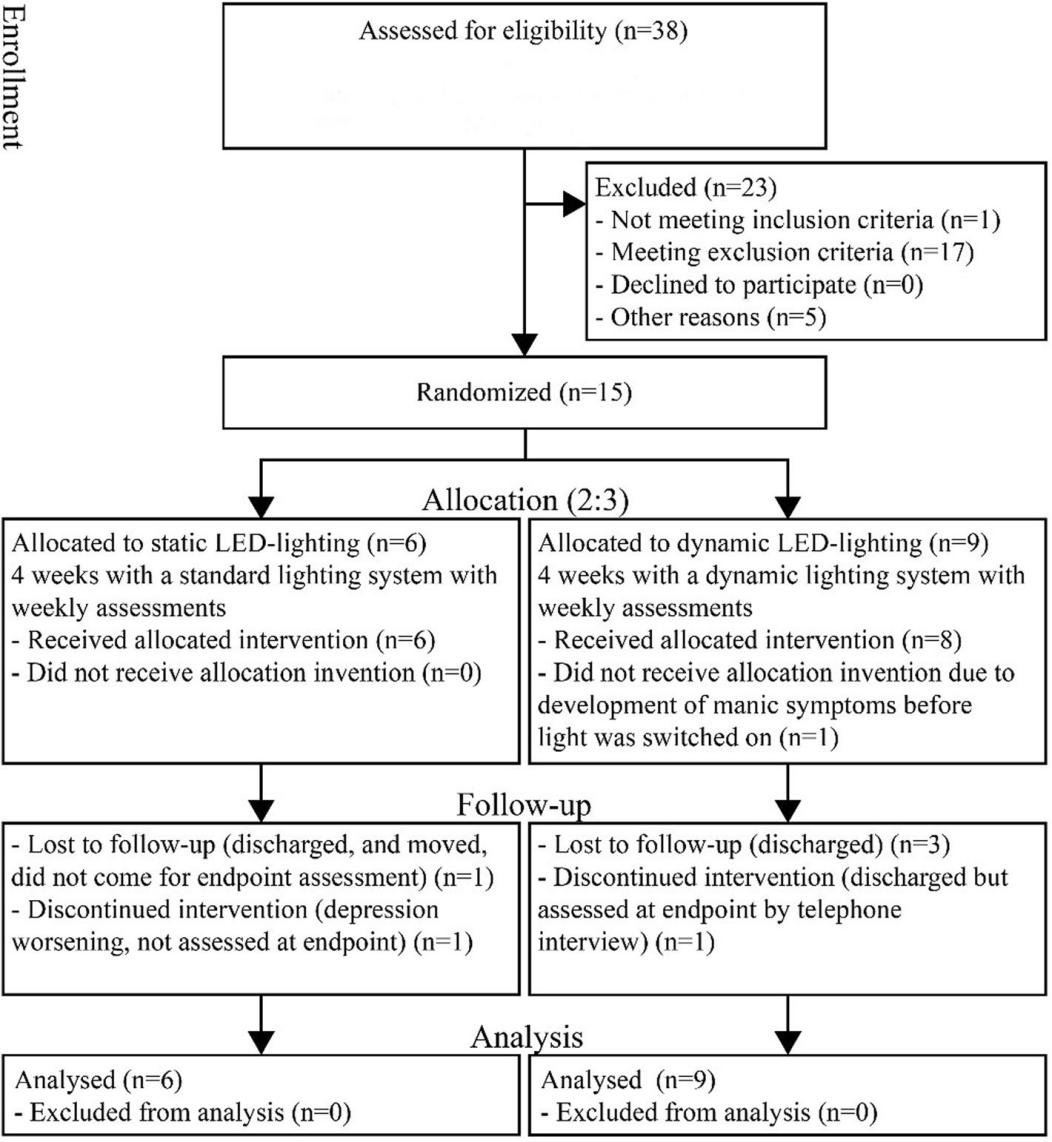
Consort flow diagram

The study was approved by the Regional Committee on Health Research Ethics (J.nr. H-17010932) and the Danish Data Protection Agency (j.nr.: VD-2018-515). Participants were assessed psychometrically weekly. All four rooms could be switched to either dynamic or static mode through a centrally placed control panel. All participants signed informed consent forms before enrollment into the study. The CONSORT flow diagram is show below in Fig. 1.

### Light intervention

**Fig. 2:**
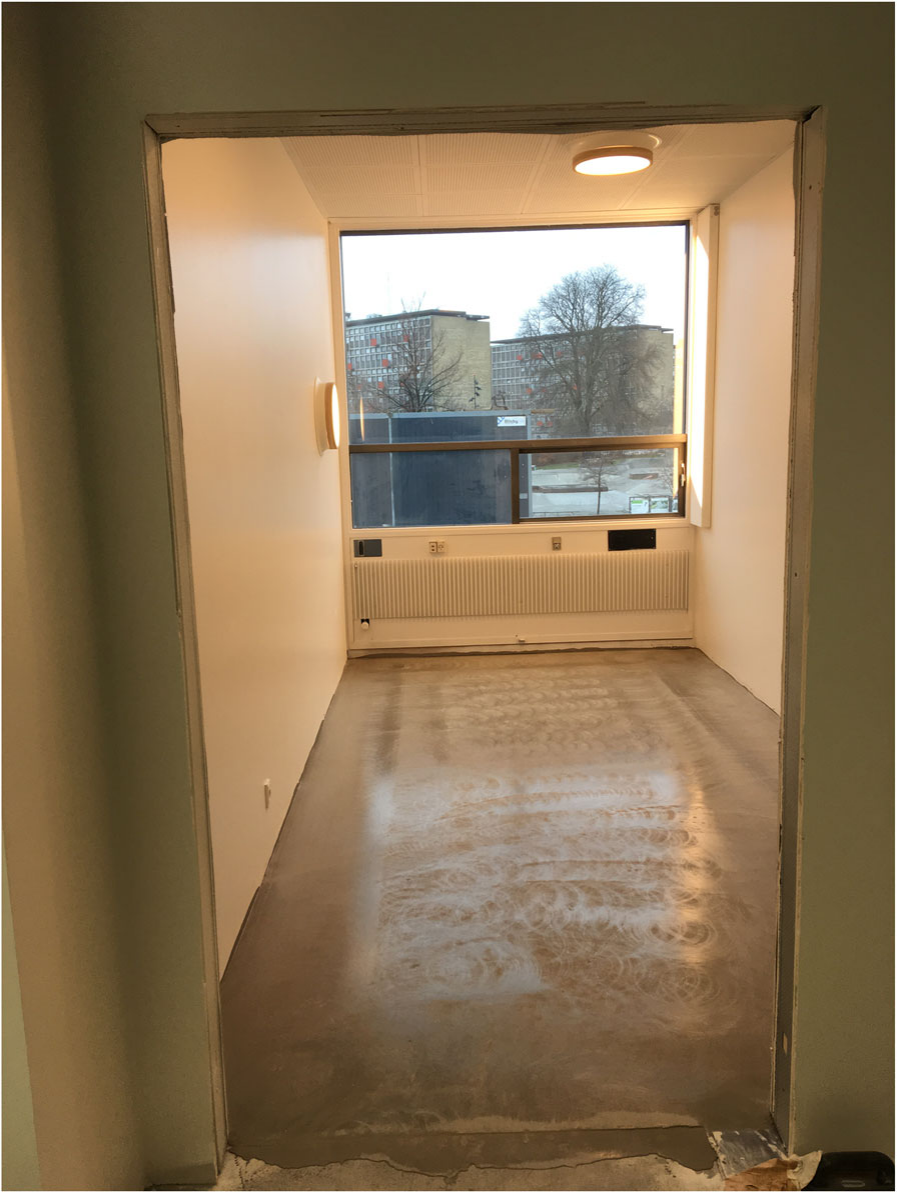
Light installation in a single-bed patient room with light in dynamic mode

The dynamic intervention setup consisted of three lighting elements (A, B and C). A was an LED luminaire built into the window jamb mimicking the natural sunlight (see Fig. 2). It was scheduled to turn on at 06:00 till 18:00 in the summer period (from February 14 to October 31) and from 07:00 till 17:00 in the winter period (from November 1 to February 14). The light from this luminaire varied continuously in Correlated Colour Temperature (CCT) from 1800 K dim, warm light at dawn rising to 5500 K bright white light from 9:00 to 14:00. From 14:00 and onward, the light from the luminaire was reduced in both intensity and CCT. The LED luminaire (A) could not be controlled by the participants, but a semi-translucent curtain could be drawn to reduce intensity. The panel contained cool white (CW), warm white (WW) and wide-spectrum amber (Amber) LEDs. B was two circular luminaires containing CW/WW/Amber LEDs mounted in the ceiling with dynamic regulation of intensity and CCT during the whole 24-h day. The light varied from 1800 K dim, warm light to 4000 K at a maximum intensity brighter than usual in a patient room. The ceiling luminaires could be turned off/on by the participants as preferred. During the summer, the dynamic LED lighting was brightest between 09:00 and 14:00 and dimmest and warmest from 23:00 to 06:00. In the winter period, the timings were changed from 09:30 to 13:30 and from 22:30 to 07:00, respectively.

C was a reading luminaire by the bed, with similar design and timing as the ceiling lighting and regulation of CCT from 1800 to 4000 K, also containing CW/WW/Amber LEDs. The intensity of the reading luminaire was kept relatively low yet permitting reading. Like the ceiling luminaire, the reading luminaire could be turned off/on by the participants as preferred. All transitions in A, B and C were made as slow, continuous fades to mimic the nature of daylight. If turned on during nighttime, the ceiling (B) and reading luminaires (C) gave a dim level Amber light. If turned off, The A, B and C luminaire started automatically in the morning with dim Amber light. In emergency situations, the dynamic regulation could be overridden to the standard (static) regime by a 3-s push on the switch.

Figure 2 shows the light installation in a patient room with the dynamic setup turned on. The window luminaire is seen built into the righthand window jamb and one on the ceiling, and the reading luminaire is also visible.

The static intervention used the same luminaires as the dynamic intervention but was completely static with regards to intensity, CCT and timing. The window jamb luminaire (A) was turned off, and the ceiling luminaire (B) and reading luminaire (C) were both set to 3000 K, with an intensity expected in a standard patient room. Both ceiling (B) and reading luminaires (C) could be turned on/off as preferred by the participants. The use of ceiling and reading luminaire was logged continuously by the computer regulating the lighting system in both groups. All participants were offered psychopharmacological, psychotherapeutic and other treatments as usual. Patients were informed that the trial tested two different lighting systems to evaluate visual comfort, lighting system performance and any influence on depressive symptoms. They were also informed that the window luminaire was not active in the static group. Participant were informed that it was not known a priory what setup would be best. Participants were thus not blinded to the intervention.

### Outcomes

The primary outcome was the rate of participants discontinuing the trial due to discomfort from the lighting conditions. Secondary outcomes were recruitment rate, dropout rate, visual comfort, level of depressive symptoms and suicidal ideation. The primary outcome was chosen because we were uncertain on how patients would react to a built-in luminaire with higher than usual intensity and a window luminaire that could not be turned off. This information is considered of major importance regarding the upcoming efficacy trial. The secondary outcomes were chosen as feasibility (dropout, visual comfort) and safety measures (suicidal ideation and depressive symptoms) and to inform about dimensioning of the upcoming efficacy trial regarding the number of rooms with light installation and trial length (recruitment flow).

Explorative outcomes were manic symptoms, self-assessed depression symptoms, quality of life, sleep, adverse effect, chronotype, room occupancy, use of luminaires, light intensity and spectral intensity, and medication. Hamilton rating was done by an experienced research coordinator that were blinded for treatment allocation and who did not have any other association with the study procedures.

### Outcome measures

Visual comfort was assessed by a newly designed Visual Comfort Scale, depression severity by the Hamilton Depression Rating Scale (HAM-D_17_) [22], and suicidal ideation by the Suicidal Ideation Attribution Scale (SIDAS) [23]. Manic symptoms were assessed by the Young Mania Rating Scale (YMRS) [24], self-assessed depression level by the Major Depression Inventory (MDI) [25], quality of life by the WHO-5 well-being index [26], sleep by the Pittsburg Sleep Quality Index (PSQI) [27] and from a diary with sleep onset and offset, naps, sleep quality and awakening. Adverse effect were assessed by the UKU (Udvalget for Kliniske Undersøgelser) adverse event scale [28], and chronotype by the Morningness-Eveningness Scale (MEQ) [29], and room occupancy from a diary estimating hours spent in the room during daytime. The use of ceiling luminaires and reading luminaires was registered (logged by the lighting computer system) throughout the study for both treatment conditions. Light sensors were mounted in each room, one at the window pointing outwards and two in the room, all measuring the intensity and spectral distribution. Medication was recorded from case files at baseline.

## Results

### Sociodemographics

Participants were recruited from December 12, 2017, to December 19, 2018. Participants had a median age [range] of 36.0 [26–56]/59.0 [34–70] years in the dynamic/static group with 66.7% females and 33.3% males in both groups. Median number (interquartile range IQR) of previous depressive episodes was 1.0 (0–2.0)/1.5 (0.5–19.0), and median duration of current depressive episode was 1.8 (1.0–8.5)/13.0 (4–41) months. In all, 20% (*n* = 3) of participants had a depressive episode as part of a bipolar disorder, see Table 1 for sociodemographic data.

**Table 1:** Sociodemographics

Parameter	Static LED-lighting group	Dynamic LED-lighting group
Participants (number of participants (percentage))	6 (40.0%)	9 (60.0%)
Gender (female/male) (number of participants (percentage))	4/2 (66.7%/33.3%)	6/3 (66.7%/33.3%)
Smoking (number of participants (percentage))	4/5 (80.0%)	3/8 (37.5%)
Self-perceived as a light sensitive person (number of participants (percentage))	2/5 (40.0%)	3/8 (37.5%)
Migraine illness (number of participants (percentage))	1/5 (20.0%)	0/8 (0%)
Eliciting factor for current depressive episode (number of participants (percentage))	3/5 (60.0%)	5/8 (62.5%)
Suicide attempt in current depressive episode (number of participants (percentage))	2/5 (40.0%)	1/8 (12.5%)
Age, years (median (IQR))	59.0 (34–70)	36.0 (26–56)
Number of previous depressive episodes (median (IQR))	1.5 (0.5–19.0)	1.0 (0–2.0)
Duration (months) of actual depressive episode (median (IQR))	13.0 (4–41)	1.8 (1.0–8.5)

*IQR* interquartile range

### Primary and secondary outcomes

Nine participants were randomised to the dynamic group and six to the static group in the period from December 12, 2017, to December 19, 2018. No participants discontinued the trial due to discomfort from the lighting condition. Recruitment rate was only 39.5% (15/38). Recruitment rate was low due to long staying non-eligible patients in one of the four test rooms. Dropout rates from treatment at week four were 55.6% (five out of nine) in the dynamic and 33.3% (two out of six) in the static group, primarily caused by early discharge (*n* = 6). Visual comfort results are shown in Table 2. Participants in the static group reported that they used the ceiling and bed luminaires to a higher degree in the morning and afternoon than the participants in the dynamic group. More glares were reported from the ceiling and reading luminaire in the dynamic group. Participants in both groups reported low satisfaction with the reading luminaires in the evening due to too low light intensity making reading difficult. Finally, some participants reported glare from the window jamb luminaire in the morning. General satisfaction with the lighting (Question 3c) was 78.1% in the dynamic group versus 71.8% in the static group. The HAM-D_17_ scale showed moderate depression levels at baseline without any difference between groups during the 4-week period. The SIDAS scale showed low to moderate degrees of suicidal ideation in both groups with baseline SIDAS scores in the dynamic/static group of 15.5 (SD = 6.8)/16.6 (SD = 11.7) and endpoint scores of 16.8 (SD = 10.4)/16.3 at (SD = 14.9) (*n* = 4/4).

**Table 2:** Visual Comfort Scale evaluated morning, afternoon and evening shown as mean percentage for all 4 weeks

Questions for all participants	Static LED-lighting group			Dynamic LED-lighting group		
	6.00 to 12.00	12.00 to 18.00	18.00 to 24.00	6.00 to 12.00	12.00 to 18.00	18.00 to 24.00
Ceiling light	Mean percentage for 4 weeks					
1a. Do you use the ceiling light? Percentage, *n*	92.3% (12/13)	92.3% (12/13)	92.3% (12/13)	63.2% (12/19)	68.8% (11/16)	87.5% (14/16)
1b. Are you satisfied with the ceiling light? Percentage, *n*	100% (13/13)	100% (13/13)	100% (13/13)	84.2% (16/19)	100% (16/16)	66.7% (10/15)
1c. Do you experience glare from the ceiling light? Percentage, *n*	7.7% (1/13)	7.7% (1/13)	15.4% (2/13)	21.1% (4/19)	12.5% (2/16)	6.3% (1/16)
Bed reading light	Mean percentage for 4 weeks					
2a. Do you use your bed reading light? Percentage, *n*	84.6% (11/13)	76.9% (10/13)	100% (13/13)	63.2% (12/19)	62.5% (10/16)	100% (16/16)
2b. Are you satisfied with the bed reading light? Percentage, n	66.7% (8/12)	33.3% (4/12)	23.1% (3/13)	63.2% (12/19)	80.0% (12/15)	43.8% (7/16)
2c. Do you experience glare from the bed reading light? Percentage, *n*	0% (13/13)	0% (13/13)	0% (13/13)	26.3% (5/19)	0% (0/16)	6.3% (1/16)
General satisfaction	Mean percentage for 4 weeks					
3a. Are you, in general, satisfied with the color of the lighting in your bedroom? Percentage, *n*	100% (13/13)	100% (13/13)	100% (13/13)	100% (19/19)	93.8% (15/16)	68.8% (11/16)
3b. Are you, in general, satisfied with the intensity of the light? Percentage, *n*	76.9% (10/13)	46.2% (6/13)	38.5% (5/13)	63.2% (12/19)	87.5% (14/16)	37.5% (6/16)
3c. Are you, in general, satisfied with the lighting in your room? Percentage, *n*	92.3% (12/13)	61.5% (8/13)	61.5% (8/13)	84.2% (16/19)	93.8% (15/16)	56.3% (9/16)
3d. Do you experience the lighting as homely and comforting? Percentage, *n*	61.5% (8/13)	61.5% (8/13)	61.5% (8/13)	38.9% (7/18)	60.0% (9/15)	73.3% (11/15)
Supplementary questions for participants in the dynamic LED-lighting group only	Mean percentage for 4 weeks					
4a. Are you satisfied with the light coming from the light panel that is built into the side of the window? Percentage, *n*	–	–	–	77.8% (14/18)	86.7% (13/15)	81.8% (9/11)
4b. Do you experience glare from the light panel built into the side of the window? Percentage, *n*	–	–	–	16.7% (3/18)	0% (0/15)	9.1% (1/11)

### Exploratory outcome measures

The mean YMRS scores were low with baseline scores of 2.2 (SD = 3.4)/0 (SD = 0) (*n* = 9/6) in the dynamic/static groups and endpoint scores of 0 (SD = 0)/0.5 (SD = 1.0) (*n* = 4/4). The baseline MDI scores in the dynamic/static groups were 29.4 (SD = 12.8)/28.0 (SD = 15.4) (*n* = 5/8), and endpoint scores were 18.8 (SD = 10.9)/27.8 (SD = 13.6) (*n* = 4/4). The baseline WHO-5 scores (highest = best) in the dynamic/static groups were 29.5 (SD = 24.2)/21.6 (SD = 8.3) (*n* = 8/5) and endpoint scores were 40.0 (SD = 27.9)/37.0 (SD = 31.7 (*n* = 4/4). Adverse events from the UKU scale were few, including low levels of headache and light sensitivity. One participant in the dynamic group had a non-serious self-harm episode, and another participant in the dynamic group, when on leave, took an overdose of medication with no complications. The last incident was reported to the research ethics committee. Neither of the incidents was considered related to the LED lighting.

Mean sleep onset, in the dynamic/static group, was 23:25 (SD = 41 min)/23:02 (SD = 52 min) during week 1 (mean of 7 days) and 23:59 (SD = 89 min)/23:29 (SD = 50 min) during week 4. Mean sleep offset in the dynamic/static groups was 6:45 (SD = 131 min)/7:18 (SD = 60 min) during week 1 and 7:46 (SD = 129 min)/7:16 (SD = 56 min) during week 4. Mean subjective sleep quality in the dynamic/static group (0–10, 10 best) was 6.5 (SD = 3.1)/6.2 (SD = 2.6) during week 1 and 7.1 (SD = 2.5)/5.2 (SD = 2.0) during week 4. There was no difference between groups in number of awakenings. Mean PSQI global scores in the dynamic/static groups (lower score = best) was 10.6 (SD = 5.2)/8.4 (SD = 5.7) at baseline and 9.0 (SD = 7.0)/13.3 (SD = 2.2) at endpoint. Mean MEQ scores showed participants to be intermediate chronotypes with baseline scores in the dynamic/static groups of 48.8 (SD = 7.6)/46.2(SD = 12.0) and endpoint scores of 46.0 (SD = 8.0)/49.8 (SD = 9.2). The room occupancy diary showed that participants, during the 4 weeks period, were in their room in the dynamic/static group for 2.3 (SD = 1.5)/2.3 (SD = 1.4) hours in the morning, 2.6 (SD = 1.7)/3.3 (SD = 1.8) hours in the afternoon and 2.2 (SD = 1.6)/3.1 (SD = 3.8) hours in the evening, summing up to 7.1 (SD = 4.1)/8.7 (SD = 3.8) hours in total. Nearly all participants used their curtains at some time of the day (100%/93.3%). However, only 15.8% used curtains all day in the dynamic group compared to 35.7% in the static group.

Luminaire log data were available for five participants in the dynamic group and four participants in the static group. The mean daytime use of the ceiling luminaires in the dynamic/static groups (07:00–23:00) was 9.2 (SD = 5.8)/3:6 (SD = 4.4) hours with a distribution of 33.3%/35.9% in the morning (7:00–12:00), 34.2%/31.8% in the afternoon (12:00–18:00) and 32.4%/32.2% in the evening (18:00–23:00). The mean nighttime (23:00–7:00) use of the ceiling luminaires in the dynamic/static groups was 81.9 (SD = 96.8)/0.7 (SD = 4.9) minutes. The mean daytime use of the reading luminaires in the dynamic/static group was 9.2 (SD = 5.4)/5.2 (SD = 4.5) hours, with a distribution of 31.0%/28.2% in the morning, 31.9%/24.6% in the afternoon and 37.2%/47.2% in the evening. The mean nighttime use of the reading luminaires in the dynamic/static groups was 86.7 (SD = 96.7)/33.5 (SD = 76.0) minutes. The LED-lighting system was 100% reliable. One participant experienced a 3-hour dark-out of the system due to a general electrical malfunction at the hospital. The light sensor system had too low sensitivity to measure the spectral distribution of room light reliable. Case files showed that participants in both groups were treated with antidepressants, mood stabilisers, antipsychotics, anxiolytics and sleeping medications.

**Fig. 3:**
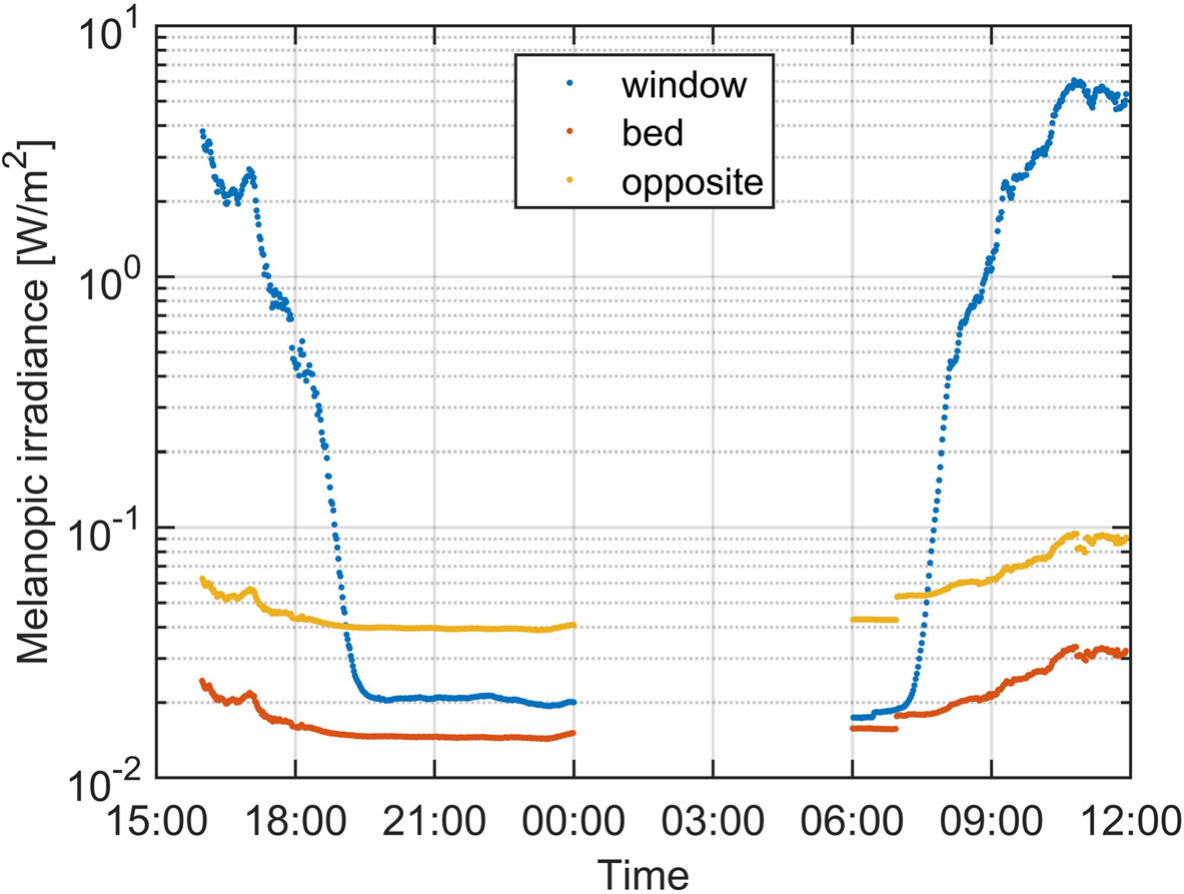
Measurement of melanopic irradiances from a sensor in the window pointing outwards (blue), a sensor above the bed (red) and a sensor opposite the bed (yellow), over a single day

Figure 3 shows an example of temporal melanopic irradiance (according to the CIE S026) measurements from a single day. The blue line is irradiance from the window daylight sensor pointing outwards. The red line is from the room sensor placed above the bed, and the yellow line is from the room sensor placed opposite the bed.

## Discussion

It was possible to evaluate the feasibility of the LED lighting system and the study design. In this study, we chose the rate of discontinuation as the primary outcome because we were unsure of how patients would react to having light treatment built into their room. We feared that too high light intensity or glare would make patients leave their room, and we also were unsure how patients would react to the dynamic luminaire built into the window jamb that they could not turn off. However, the results showed no discontinuation due to discomfort from the lighting system, and the satisfaction with the dynamic system was equal or higher than with the static system. There were a low recruitment rate and large attrition rate, especially at week four. Most participants in both groups were satisfied with the lighting system. Depressive symptoms were moderate at baseline. The high dropout from treatment at endpoint made outcomes difficult to interpret. The high dropout was primarily due to early discharge. There was a moderate degree of suicidal ideation in both groups.

Methodological strengths include a randomised design, reaching the sample size, a detailed assessment of feasibility measures and functionality of the lighting system.

Methodological limitations include a low sample size with an inherently large risk of type II errors, large dropout and a malfunctioning lighting sensor system. The imbalance between groups in the mean length of current depressive episodes is likely an artefact of the low sample size.

## Conclusion

A larger number of light-equipped rooms would help attain a higher number of recruitments per month, making larger trials more feasible. The study period should be shortened to reduce dropout due to early discharge. The most important is to secure an endpoint assessment, even if the participants drop out due to early discharge or for other reasons. It may induce bias if trial participants are not assessed at follow-up, and we should aim at a 95% completion rate. This completion rate might be accomplished by informing in the oral presentation and in the written material that an endpoint assessment is important even if the participant is discharged. The visual comfort data indicated that light intensity was too low in the evening, and pointing to that in future trials, intensity of light can be increased. Higher light intensity should, however, contain very little blue light to avoid undue melatonin suppression that would disturb sleep. The light sensor system should be replaced with more sensitive and stable sensors. The study did show that it was possible to make a detailed assessment of patients regarding feasibility and depression and sleep outcomes. The study shows the importance of carrying out a feasibility trial to test the design and technical systems and to evaluate lighting tolerability. We are looking forward to implementing these results in a randomised clinical efficacy trial with dynamic LED lighting.

This efficacy trial (now running) has ten rooms (out of 12 rooms) installed with a similar lighting system in a similar ward. We believe this will increase the number of inclusions. The efficacy trial aims at a total of 150 patients, with 75 in each group. This larger sample will increase the power to detect clinically meaningful differences between groups. We have decided to reduce the trial length from 4 to 3 weeks, thus reducing attrition due to early discharge. Furthermore, we have implemented a mandatory endpoint assessment at week 3 for all included patients even if they are discharged early or if the intervention is stopped for other reasons.

## Data Availability

The datasets that was generated and analysed during the current study are not publicly available due to Danish data protection rules, but are available from the corresponding author on reasonable request.

## Abbreviations

CCT: Correlated Colour Temperature
CTU: Copenhagen Trial Unit
CW: Cool White light-emitting diodes
DSM-IV: Diagnostic and Statistical Manual of Mental Disorders, IV version
HAM-D_17_: Hamilton Depression rating scale 17-item version
eCRF: electronic Case Report Form
ipRGC: Intrinsically photosensitive Retinal Ganglion Cells
LED: Light-emitting diodes
MEQ: Morningness-Eveningness Questionnaire
M.I.N.I.: Mini International Neuropsychiatric Interview
OpenClinica: An electronic case report system, including randomization and data capture
PSQI: Pittsburgh Sleep Quality Index
SIDAS: Suicidal Ideation Attributes Scale
UKU: The UKU adverse event rating scale (acronym for Udvalget for Kliniske Undersøgelser)
WHO: World Health Organization
YMRS: Young Mania Rating Scale
WW: Warm white light-emitting diodes
